# Effects of Type 2 Diabetes on the Neuropsychological Profile in Mild Cognitive Impairment

**DOI:** 10.3233/JAD-230791

**Published:** 2024-05-28

**Authors:** José A. Reyes Bueno, Guillermo Sánchez-Guijo, Pablo Doblas Ráez, Juan A. García-Arnés, Francisco J. Garzón-Maldonado, Vicente Serrano Castro, Carlos de la Cruz-Cosme, Carmen Alba-Linero, Mario Gutiérrez-Bedmar, Natalia García-Casares

**Affiliations:** a Departamento de Neurología, Hospital Universitario Regional de Málaga, Málaga, Spain; b Departamento de Medicina, Facultad de Medicina, Universidad de Málaga, Málaga, Spain; c Departamento de Neurología, Hospital Universitario Virgen de la Victoria de Málaga, Málaga, Spain; d Instituto de Investigación Biomédica de Málaga y Plataforma en Nanomedicina-IBIMA Plataforma BIONAND, Málaga, Spain; e CIBERCV Cardiovascular Diseases, Carlos III Health Institute, Madrid, Spain; f Departamento de Oftalmología, Hospital Universitario Virgen de la Victoria de Málaga, Málaga, Spain; g Centro de Investigaciones Médico-Sanitarias (CIMES), Spain

**Keywords:** Alzheimer’s disease, cognition, diabetes mellitus, mild cognitive impairment, neuropsychological test, type 2 diabetes

## Abstract

**Background:**

Diabetes is one of the main risk factors for developing mild cognitive impairment (MCI) and Alzheimer’s disease. Most studies have demonstrated a worse performance in executive function, verbal fluency, and information processing speed in patients with diabetes.

**Objective:**

To assess the cognitive functioning of persons with type 2 diabetes and amnesic mild cognitive impairment (aMCI-T2DM) compared to persons with aMCI without diabetes and persons without diabetes or aMCI as controls, to understand the role of diabetes in the neuropsychological profile.

**Methods:**

Cross-sectional study involving a sample of 83 patients, ranging in age from 61 to 85 years and divided into three groups: aMCI-T2DM (27 patients), aMCI (29 patients), Controls (27 individuals). All the participants undertook an exhaustive neuropsychological assessment (auditory-verbal and visual memory, attention, information processing speed, language, executive function, and depression).

**Results:**

Both groups of aMCI patients performed significantly worse than the controls in all the neuropsychological tests. A significant linear tendency (*p* trend < 0.05) was found between groups, with the aMCI-T2DM group presenting worse results in global cognition assessed by the Mini-Mental State Examination and Montreal Cognitive Assessment; Rey-Osterrieth Complex Figure Test; Auditory Verbal Learning Test; Trail Making Test A and B, Verbal Fluency Test, and Hamilton Depression Rating Scale.

**Conclusions:**

aMCI patients with or without diabetes showed worse cognitive function compared to persons without diabetes or aMCI. Additionally, aMCI patients without T2DM presented a different cognitive profile than aMCI patients with T2DM, which tended towards presenting worse cognitive functions such as global cognition, memory, attention, executive function, and language.

## INTRODUCTION

Type 2 diabetes mellitus (T2DM) is a metabolic disorder defined by chronic hyperglycemia due to insulin resistance and a variable level of defective secretion of this hormone. T2DM is one of the most prevalent chronic diseases. In 2021, the estimated prevalence of T2DM in people aged 20 to 79 years was 10.5% of the global population (536.6 million people worldwide) [1]. The maximum prevalence of T2DM is set between the ages of 65 and 79. An increase in the prevalence of diabetes mellitus is expected because of lifestyle changes, higher life expectancy and population aging [2].

Dementia is a syndrome characterized by a decline in memory and intelligence, behavioral changes, and an impairment for activities of daily living. Approximately 50 million people suffer from dementia globally and this number is expected to rise to 139 million by 2050 [3]. The prevalence of mild cognitive impairment (MCI) in the population over 65 years of age is estimated to be around 3–22%. In addition, a higher risk of developing dementia has been reported in people with MCI (5–10% increased risk per year) compared to the general population (1–2% increased risk per year) [4].

Different studies [5, 6] have shown that T2DM is one of the main risk factors for developing MCI and Alzheimer’s disease (AD). Diabetes mellitus increases the probability of developing dementia, including AD, by two to three times [7]. An association between T2DM and MCI is frequent. The prevalence of MCI in T2DM patients is estimated to be 45%, ranging from 21.8% to 67.5% depending on the study [4].

Although the link between these two pathologies is already known, the underlying pathophysiology of cerebral neurodegeneration and its relationship with diabetes is not yet fully understood. Nonetheless, insulin resistance, hyperinsulinemia, hyperglycemia, inflammatory agents, and genetic factors are all thought to be involved in *β*-amyloid and tau protein deposition in the brain [7–11]. The hippocampus, entorhinal cortex, and frontal cortex, among other areas, are cerebral regions with numerous insulin receptors, which makes them vulnerable to cognitive impairment due to chronic hyperinsulinemia [7, 9–11]. A better description of the pathophysiological mechanisms could clarify the etiology of dementia, leading to the development of new treatments [7].

Previous studies have compared patients with both MCI and T2DM with controls, with the former group obtaining worse results in the neuropsychological tests. However, the results regarding the affected domains differ depending on the study, possibly due to the methodological diversity (study design, sample size and usage of different neuropsychological tests). Most studies demonstrated a worse performance in executive function, verbal fluency, and information processing speed [12–14].

There is also evidence that non-amnestic forms of MCI predominate in patients with diabetes, which has a lower risk of progression to AD than the amnestic subtype [9].

The objective of this study was to analyze the neuropsychological profile of patients with diabetes plus MCI versus patients with just amnestic MCI (aMCI) and compare the results with persons without diabetes or aMCI (controls), to elucidate the role of diabetes in neurodegeneration. We hypothesized that aMCI-T2DM subjects would have a poorer performance when compared to controls and a different neuropsychological profile in comparison with aMCI patients without T2DM.

## MATERIALS AND METHODS

### Study design and patients

We undertook a cross-sectional study with a sample of 83 patients (aged 61 to 85 years) recruited from the outpatient dementia and diabetes mellitus units of the hospital. The participants were divided into three groups: aMCI-T2DM, aMCI without T2DM, and persons without diabetes or aMCI as controls. Patients were approached and recruited according to the inclusion and exclusion criteria.

### Inclusion criteria

aMCI was diagnosed based on the 2006 criteria of the European Alzheimer’s Disease Consortium. These include cognitive (memory) complaints, Montreal Cognitive Assessment (MoCA)≥26 and/or Mini-Mental State Examination (MMSE)≥24, Clinical Dementia Rating = 0.5 and no significant repercussions on activities of daily living. T2DM patients were diagnosed based on 2006 criteria of the American Diabetes Association and medical record. Additionally, T2DM patients all presented good control of the diabetes with glycosylated hemoglobin (HbA1c) levels under 7% and none were receiving insulin treatment.

### Exclusion criteria

Persons with one or more of the following conditions were excluded from the study: a) incapacity or unwillingness to sign the informed consent; b) medical record of any chronic disease or neurological condition that may affect cognitive function; c) traumatic brain injury with loss of consciousness, learning disorders or mental retardation; d) previous vascular disease (stroke or acute myocardial infarction) and/or anticoagulant therapy; e) active neoplasia or a history of neoplasia within the last five years; f) severe psychiatric disease; g) severe disease in which life expectancy is lower than 24 months; h) addiction to illegal drugs; i) microvascular complications, including nephropathy, retinopathy or neuropathy; j) left-handedness, assessed with the Edinburgh Handedness Inventory; k) magnetic resonance imaging (MRI) contraindications (pacemakers, metallic prosthesis, pregnancy, severe claustrophobia); l) T2DM patients on insulin therapy.

Additional exclusion criteria for controls were a) diagnosis of T2DM; b) cardiovascular risk factors; c) psychiatric or neurological disorders; d) treatment with any oral antidiabetic agent (including metformin, glucagon-like peptide-1 receptor agonists and sodium-glucose cotransporter-2 inhibitors); e) MoCA < 26; f) MMSE < 24.

### Variables of interest and data collection

An anamnesis, neuropsychological and psychiatric examination, and MRI were carried out in all the subjects. Medical history and physical examination were performed in all patients, and demographic data were recorded.

Educational status was classified into three groups: primary education (schooling up to 12 years), secondary education (schooling between 13–18 years), tertiary education (university studies and studies > 18 years beyond secondary education).

Neuropsychological examination included MMSE [15], MoCA [16] as a cognitive impairment screening test, Wechsler Abbreviated Scale of Intelligence [17], Rey Auditory Verbal Learning Test (RAVLT) [18], Rey-Osterrieth Complex Figure Test (RCFT) [19], Trail Making Test (TMT) [20], Verbal Fluency Test (VFT) [21], Stroop Color and Word Test [22], and Hamilton Depression Rating Scale [23]. This set of neuropsychological tests assesses intelligence, auditory-verbal and visual memory, attention and information processing speed, language, attention and executive function, and depression, respectively.

Scores in parts A and B of the TMT were based on the time needed to complete the test. The time ranges from 0 to 240 s for TMT A and from 0 to 360 s for TMT B. The highest time lapse allowed (TMT A = 240 and TMT B = 360) was given to those persons who were unable to finish the task in the established period, as established in the methods of prior studies [8].

All patients were evaluated in the same room in the facility, in the morning and under the same lighting conditions.

In addition, secondary causes of aMCI were studied in all subjects while performing MRI and normal blood test, including thyroid function, vitamin B12 levels and lues serology.

This study followed the recommendations of the Declaration of Helsinki and was approved by the ethics committee. All the participants signed the informed consent.

### Statistical analysis

Characteristics of participants are described as mean±standard deviation for continuous variables and count (percentages) for categorical variables. Group comparisons for nominal variables were carried out using the Chi-square test if all expected frequencies were≥5 or Fisher’s exact test otherwise. For numerical variables, group comparisons were made using the Kruskal-Wallis test because assumptions for using analysis of variance (ANOVA) were not met.

Mean scores of neuropsychological variables with 95% confidence intervals were estimated using ANOVA. Polynomial contrast (linear trend) was used to evaluate the progressive variation of such mean scores across groups (aMCI + T2DM, aMCI without T2DM, and controls).

All statistical tests were two-sided and *p* values < 0.05 were considered statistically significant. All statistical analyses were done using Stata 17.0 (StataCorp LLC).

## RESULTS

This study included 83 participants, 27 classified as aMCI-T2DM, 29 as aMCI without T2DM, and 27 controls without cognitive symptoms or diabetes. There were no significant differences in gender distribution (*p* = 0.062), age (*p* = 0.069), presence of dyslipidemia (*p* = 0.174), proportion of smokers (*p* = 0.403), body mass index level (*p* = 0.183), or educational level (*p* = 0.255). On the other hand, there was a significantly higher proportion of hypertensive patients in the aMCI-T2DM and aMCI groups compared to the controls (*p* = 0.029). The presence of hypertension did not differ significantly between the two aMCI groups (59.3% of aMCI-T2DM patients and 62.1% in aMCI patients without diabetes, *p* > 0.05) (see Table 1).

**Table 1:** Clinical and demographic characteristics of the study population

VARIABLE	aMCI-T2DM *n* = 27	aMCI *n* = 29	Control *n* = 27	*p*
Female sex	8 (29.6%)	17 (58.6%)	15 (55.6%)	0.062
Age	72.70±5.642	73.17±6.514	70.41±3.533	0.166
Hypertension	16 (59.3%)	18 (62.1%)	8 (29.6%)	0.029
Dyslipidemia	16 (59.3%)	11 (37.9%)	10 (37%)	0.174
Smoker	5 (18.5%)	2 (6.9%)	3 (11.1%)	0.405*
BMI (kg/m^2^)	27.47±3.367	26.74±4.201	25.49±3.916	0.130
Level of education				0.066*
Primary education	7 (25.9%)	5 (17.2%)	0 (0%)	
Secondary education	9 (33.3%)	11 (37.9%)	13 (48.1%)	
Tertiary education	11 (40.7%)	13 (44.8%)	14 (51.9%)	

Comparisons between groups were made with Chi-square test or Fisher’s exact test* (for nominal variables) and Kruskal-Wallis test (for quantitative variables). Data are expressed as absolute and relative frequencies for nominal variables and as mean±standard deviation for quantitative variables. aMCI-T2DM, mild cognitive impairment and type 2 diabetes; aMCI, amnesic mild cognitive impairment; BMI, body mass index.

**Fig. 1:**
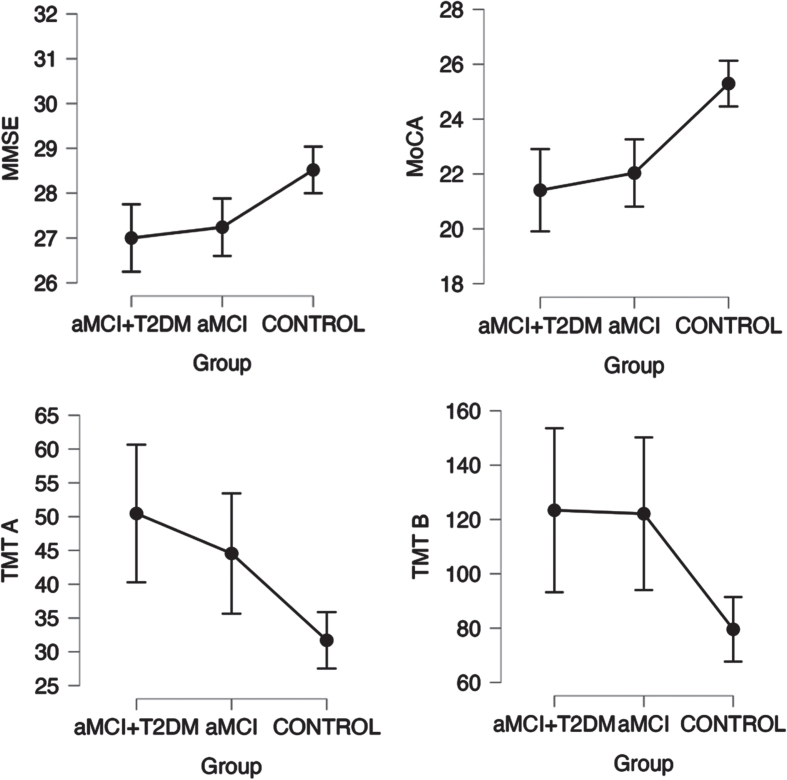
Linear trend between groups in neuropsychological tests for screening cognition (MMSE and MoCA), attention and executive function (Stroop Word and Color Test), with a statistically significant trend (*p*-trend < 0.05). Points represent mean values and bars indicate 95% confidence interval. aMCI-T2DM, amnesic mild cognitive impairment with type 2 diabetes; aMCI, amnesic mild cognitive impairment; MMSE, Mini-Mental State Examination; MoCA, Montreal Cognitive Assessment; TMT A, Trail Making Test A; TMT B, Trail Making Test B.

**Fig. 2:**
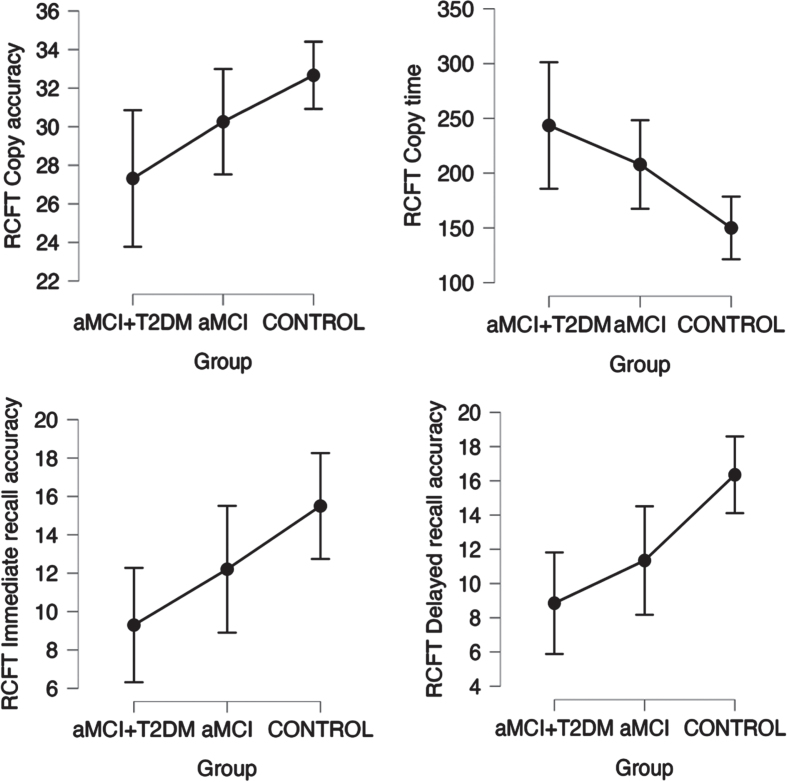
Linear trend between groups in neuropsychological tests for visual memory (RCFT Test) with a statistically significant trend (*p* trend < 0.05). Points represent mean values and bars indicate 95% confidence interval. aMCI-T2DM, amnesic mild cognitive impairment with type 2 diabetes; aMCI, amnesic mild cognitive impairment; RCFT, Rey-Osterrieth Complex Figure Test.

**Fig. 3:**
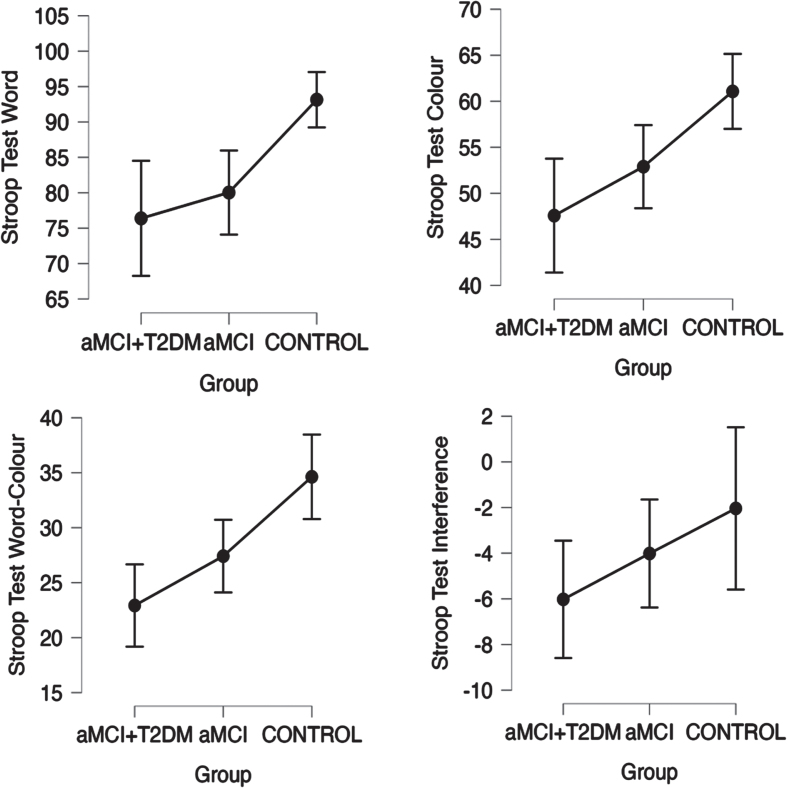
Linear trend between groups in neuropsychological tests for attention and executive function (Stroop Word and Color Tests) with statistically significant trend (*p*-trend < 0.05). Points represent mean values and bars indicate 95% confidence interval. aMCI-T2DM, amnesic mild cognitive impairment with type 2 diabetes; aMCI, amnesic mild cognitive impairment.

**Fig. 4:**
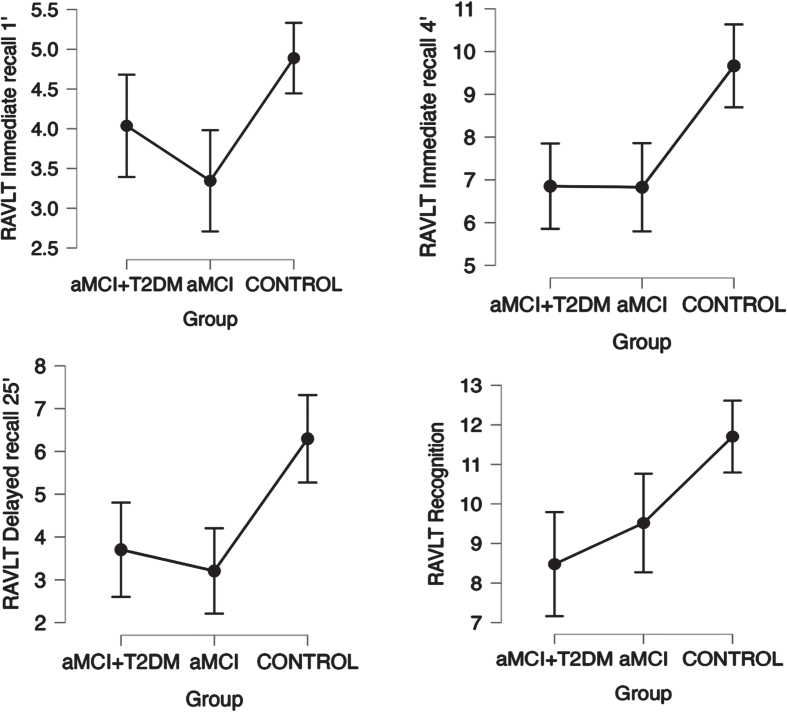
Linear trend between groups in neuropsychological tests for auditory-verbal memory (RALVT Test) with a statistically significant trend (*p* trend < 0.05) with a 95% confidence interval. Points represent mean values and bars indicate 95% confidence interval. aMCI-T2DM, amnesic mild cognitive impairment with type 2 diabetes; aMCI, amnesic mild cognitive impairment; RALVT, Auditory Verbal Learning Test.

Patients in the aMCI-T2DM group showed a trend towards worse performance on tests of global cognition (*p*-trend = 0.001 for MMSE; *p*-trend = <0.0001 for MoCA. See Fig. 1); information processing speed and executive function (*p*-trend = 0.001 for TMT-A; *p*-trend = 0.011 for TMT-B; *p*-trend = <0.001 for Stroop Test Word, Stroop Test Color, and Stroop Test Word-Color. See Fig. 2); visual memory and visuoconstructive skills (*p*-trend = 0.003 for RCFT copy time; *p*-trend = 0.007 for RCFT copy accuracy; *p*-trend = 0.005 for RCFT immediate recall accuracy; *p*-trend = <0.001 for RCFT delayed recall accuracy. See Fig. 3); verbal fluency (*p*-trend = <0.001 for VFT letter P; *p*-trend = <0.001 for VFT letter M; *p*-trend = <0.001 for VFT letter R; *p*-trend = <0.001 for VFT total words and *p*-trend = <0.001 for VFT animals. See Fig. 4); and depression (*p*-trend = <0.001 for Hamilton Depression Rating Scale).

In verbal memory, the aMCI-T2DM and the aMCI without diabetes performed similarly in the RALVT (see Fig. 4).

Table 2 shows the differences between the three groups in relation to their performance in the neuropsychological tests.

**Table 2:** Neuropsychological assessment results between groups: aMCI with type 2 diabetes; aMCI without diabetes, and Controls

Variable	aMCI-T2DM	aMCI	Control	F	*p* for linear trend
MMSE	27.00 (26.37, 27.63)	27.24 (26.63, 27.85)	28.52 (27.89, 29.15)	6.64	0.001
MoCA	21.41 (20.21, 22.60)	22.03 (20.88, 23.19)	25.30 (24.10, 26.49)	12.13	< 0.001
RCFT
Copy time	243.52 (200.58, 286.46)	207.90 (166.46, 249.33)	150.00 (107.06, 192.94)	4.79	0.003
Copy accuracy	27.31 (24.60, 30.03)	30.26 (27.63, 32.88)	32.67 (29.95, 35.39)	3.85	0.007
Immediate recall time	137.80 (116.14, 159.46)	134.89 (114.05, 155.73)	116.80 (95.14, 138.46)	1.10	0.176
Immediate recall accuracy	9.30 (6.31, 12.29)	12.21 (9.33, 15.09)	15.50 (12.52, 18.48)	4.29	0.005
Delayed recall time	107.82 (88.93, 126.70)	99.62 (81.85, 117.38)	93.71 (75.22, 112.20)	0.57	0.291
Delayed recall accuracy	8.85 (6.06, 11.64)	11.34 (8.56, 14.04)	16.35 (13.53, 19.14)	7.44	< 0.001
RAVLT
Immediate recall 1’	4.04 (3.46, 4.61)	3.34 (2.79, 3.90)	4.89 (4.31, 5.46)	7.41	0. 040
Immediate recall 4’	6.85 (5.87, 7.84)	6.83 (5.88, 7.78)	9.67 (8.68, 10.65)	11.04	< 0.001
Delayed recall 25’	3.70 (2.68, 4.72)	3.21 (2.22, 4.19)	6.30 (5.28, 7.32)	10.66	< 0.001
Recognition	8.48 (7.30, 9.66)	9.52 (8.43, 10.61)	11.70 (10.61, 12.80)	8.50	< 0.001
STROOP TEST
Word	76.38 (70.25, 82.52)	80.03 (74.23, 85.84)	93.15 (87.13, 99.17)	8.45	< 0.001
Color	47.57 (42.65, 52.50)	52.90 (48.23, 57.56)	61.07 (56.24, 65.91)	7.72	< 0.001
Word, Color	22.92 (19.32, 26.52)	27.41 (24.01, 30.82)	34.63 (31.10, 38.16)	10.91	< 0.001
Interference	–6.02 (–8.86, –3.18)	–4.01 (–6.70, –1.32)	–2.04 (–4.83, 0.75)	1.98	0.050
TMT
TMT A	50.47 (42.41, 58.53)	44.55 (36.78, 52.33)	31.70 (23.65, 39.76)	5.62	0.001
TMT B	123.39 (98.19, 148.59)	122.12 (100.01, 148.59)	79.56 (57.86, 101.25)	4.95	0.011
VFT
Letter P	11.44 (9.87, 13.02)	12.62 (11.10, 14.14)	15.70 (14.13, 17.28)	7.72	< 0.001
Letter M	10.07 (8.45, 11.70)	12.00 (10.43, 13.57)	14.41 (12.78, 16.04)	7.04	< 0.001
Letter R	9.96 (8.48, 11.44)	11.28 (9.85, 12.70)	14.22 (12.74, 15.70)	8.64	< 0.001
Total words	31.48 (27.28, 35.68)	35.86 (31.81, 39.92)	44.33 (40.13, 48.54)	9.59	< 0.001
Animals	13.63 (12.17, 15.09)	13.76 (12.35, 15.17)	18.78 (17.32, 20.24)	16.22	< 0.001
WASI	36.96 (31.43, 42.50)	39.14 (33.80, 44.48)	51.07 (45.54, 56.60)	7.52	< 0.001
HAMILTON DEPRESSION	6.41 (4.75, 8.07)	4.93 (3.33, 6.53)	1.63 (0.00, 3.29)	8.60	< 0.001
RATING SCALE

Data are expressed as mean and 95% confidence interval in parentheses. aMCI-T2DM, mild cognitive impairment and type 2 diabetes; MCI, mild cognitive impairment; MMSE, Mini-Mental State Examination; MoCA, Montreal Cognitive Assessment; RCFT, Rey Osterrieth Complex Figure Test; RAVLT, Rey Auditory Verbal Learning Test; TMT A, Trail Making Test A; TMT B, Trail Making Test B; VFT, Verbal Fluency Test; WASI, Wechsler Abbreviated Scale of Intelligence.

## DISCUSSION

The aim of this study was to compare the neuropsychological profile of patients with both MCI plus T2DM and patients with just MCI without T2DM and compare these with cognitively healthy persons without diabetes. We found that the aMCI plus T2DM group performed worse than controls in the all the tests, and additionally presented a different cognitive pattern with respect to the aMCI group without diabetes.

Our study shows that patients with both aMCI and diabetes tend to have a worse cognitive performance in global cognition, information processing speed, attention, executive tasks, visual memory, and verbal fluency compared to patients with aMCI without diabetes. On the other hand, the two groups have a similar performance in auditory-verbal memory tasks (RAVLT).

These results are similar to those found in our previous work [7, 12]. Although only a few studies have evaluated the neuropsychological variations between aMCI-T2DM and aMCI without diabetes, our results resemble those reported in other studies about aMCI-T2DM.

The study by Zhao et al. [13] with a sample size of 7082 subjects, found that individuals with T2DM had worse executive function than patients without diabetes, as well as worse global cognition. This conclusion resembles ours. It shows that decline in executive function is one of the main features of the typical neuropsychological profile of patients with T2DM.

Palta et al. [8], in a longitudinal study, included 286 patients with diabetes compared to 2741 patients without diabetes (all of them with MCI), and found that patients with diabetes performed worse in executive function and language. However, the authors acknowledged that they did not differentiate between type 1 or type 2 diabetes or other factors such as the severity of diabetes, the presence of obesity or the formal diagnosis of diabetes.

Similar outcomes were suggested by Valenza et al. [9] in their comparative study of MCI with and without diabetes. The main finding was that executive function was more impaired in patients with diabetes. Additionally, they showed that high HbA1c levels correlated with episodic memory, concluding that the severity of T2D is associated with a greater compromise of the memory, and that milder forms of T2D may be related with an attention-executive functioning deficit. This is in line with our results as all the patients with type 2 diabetes included in our study presented good diabetes control and none of them were on insulin treatment. In our study, patients with diabetes tended to perform worse in executive functions than patients without diabetes (see Fig. 2), though this was not seen for auditory-verbal memory (see Fig. 4).

Patients with aMCI and diabetes have greater alterations in the functions of attention, retrieval capacity and speed of information processing. Impairments in memory and verbal fluency abilities may be more related to impairments in executive and attentional functions than to hippocampal dysfunction (encoding and storage problems); additionally, patients with diabetes maintain similar verbal memory functions to patients with aMCI without diabetes. This is consistent with our study and others reviewed [8, 9, 13].

In our study we also observed that patients with diabetes performed worse in tests that measure visuoconstructive function and visual memory (RCFT) and other tests that use visual information to measure other cognitive areas (TMT, Stroop test) (see Figs. 1–3). Other studies have already observed these impairments. Callisalla et al. [14] found a lower performance in visuoperceptual function and Moran [24] also reported a poorer visual memory performance.

Patients with diabetes might have greater ophthalmological alterations due to the disease itself altering the imputation of visual information, as pathological processes in the retina begin early, possibly before symptoms are noticeable [25]. This is a factor that we have tried to control for by excluding patients with diabetic retinopathy or significant visual dysfunction and ensuring that all patients used visual aids if necessary. However, we cannot completely rule out this mechanism.

The cognitive impairment pattern caused by diabetes remains unclear. Moran et al. suggested in a study including the ADNI cohort that TDM2 promotes and contributes to neurodegeneration independently of the diagnosis of AD via phosphorylation of the tau protein, supported by the lack of amyloid PET differences, but with the presence of differences in CSF tau between the diabetes/no diabetes groups [26]. This may have a structural influence with a greater regional brain atrophy (bilaterally distributed in hippocampal, temporal, frontal, and cingulate cortices and subcortical nuclei) correlated with cognitive impairment of this profile [24, 27, 28]. All of this is associated, according to the available evidence, with a greater burden of vascular pathology, neuroinflammation and disorder of brain insulin signaling, as well as alterations in certain neurotransmitter systems such as the dopaminergic and serotonin pathways in patients with diabetes that contribute to cognitive impairment and probably to its clinical profile[29–31].

Studies of brain atrophy in patients with diabetes show an increased loss of gray matter, mainly in the medial temporal, anterior cingulate, and medial frontal lobes, with white matter loss distributed in the frontal and temporal regions [24, 27, 28] A 5-year follow-up study including 705 patients (348 with T2DM) showed that patients with T2DM have greater cerebral atrophy but not a greater rate of decline, suggesting that atrophy does not mediate associations between type 2 diabetes and cognitive decline [14].

The presence of hypertension was significantly higher in the two aMCI groups compared to the control group. This suggests that hypertension is a major risk factor in the genesis of cognitive impairment, especially vascular and degenerative impairment, through different pathophysiological mechanisms [32]. However, since there was no significant difference between the aMCI-T2DM and the aMCI without diabetes groups, we consider that this factor does not significantly influence the profile of cognitive impairment in patients with aMCI and diabetes.

Another interesting factor that increases the risk of developing degenerative dementia and cognitive deterioration is depression. As in cognitive impairment, depression occurrence is two to three times higher in people with diabetes. Studies show that the presence of significant depression alters the cognitive performance of patients with MCI, especially in patients who also suffer from diabetes [31, 33]. In our study, a significant linear trend was observed in depression between groups, being worse in patients with diabetes (see Table 2).

Our study has several strengths. A set of validated tests was thoroughly designed to allow us to detect cognitive changes when carried out by the patients. All patients suffered from the same type of diabetes, which was well controlled without requiring insulin treatment. This is important since some studies show that the type of diabetes and its severity influence the clinical profile of cognitive impairment [9, 34]. Furthermore, the whole sample underwent a high-field MRI to discard potential causes of secondary cognitive impairment. Nevertheless, the study also has a few limitations. The limited sample size of our study prevents us from drawing more consistent conclusions and the results must be considered carefully because of their reduced external validity. Furthermore, causality could not be established as this was a cross-sectional study.

### Conclusions

The cognitive profile of both groups of aMCI patients (with and without diabetes) is worse with respect to the neuropsychological pattern of persons without aMCI or diabetes (controls), showing a significant linear trend in global cognition, visual memory, executive functions, attention, and language. Few studies have compared the neuropsychological differences between aMCI patients (with and without diabetes) that enable us to determine the role of diabetes in mild cognitive impairment. In our study, however, we found that in patients with aMCI, the presence of diabetes contributes to a greater impairment of executive functions, information processing speed, visual memory and verbal frequency compared to patients with aMCI without diabetes.

## AUTHORS CONTRIBUTIONS

Jose A Reyes-Bueno (Methodology; Writing – original draft; Writing – review & editing); Guillermo Sánchez-Guijo (Methodology; Writing – original draft); Pablo Doblas-Ráez (Data curation; Methodology); Juan A. García-Arnés (Conceptualization; Methodology); Francisco J Garzón-Maldonado (Data curation; Methodology); Vicente Serrano-Castro (Data curation; Methodology); Carmen Alba-Linero (Methodology; Writing – original draft); Carlos de la Cruz-Cosme (Formal analysis; Methodology); Mario Gutiérrez-Bedmar (Conceptualization; Formal analysis; Methodology; Writing – review & editing); Natalia García-Casares (Conceptualization; Funding acquisition; Methodology; Supervision; Writing – review & editing).

## Data Availability

The data supporting the findings of this study are available on request from the corresponding author. The data are not publicly available due to privacy or ethical restrictions.
